# Antioxidant Activity of Oat Proteins Derived Peptides in Stressed Hepatic HepG2 Cells

**DOI:** 10.3390/antiox5040039

**Published:** 2016-10-20

**Authors:** Yichen Du, Ramak Esfandi, William G. Willmore, Apollinaire Tsopmo

**Affiliations:** 1 Food Science and Nutrition, Department of Chemistry, Carleton University, Ottawa, ON K1S 5B6, Canada; YichenDu@cmail.carleton.ca (Y.D.); RamakEsfandi@cmail.carleton.ca (R.E.); 2 Department of Biology and Institute of Biochemistry, Carleton University, Ottawa, ON K1S 5B6, Canada; Bill.Willmore@carleton.ca

**Keywords:** oat peptides, cytoprotection, antioxidant enzymes, HepG2 cells

## Abstract

The purpose of this study was to determine, for the first time, antioxidant activities of seven peptides (P1–P7) derived from hydrolysis of oat proteins in a cellular model. In the oxygen radical absorbance capacity (ORAC) assay, it was found that P2 had the highest radical scavenging activity (0.67 ± 0.02 µM Trolox equivalent (TE)/µM peptide) followed by P5, P3, P6, P4, P1, and P7 whose activities were between 0.14–0.61 µM TE/µM). In the hepatic HepG2 cells, none of the peptides was cytotoxic at 20–300 µM. In addition to having the highest ORAC value, P2 was also the most protective (29% increase in cell viability) against 2,2′-azobis(2-methylpropionamidine) dihydrochloride -induced oxidative stress. P1, P6, and P7 protected at a lesser extent, with an 8%–21% increase viability of cells. The protection of cells was attributed to several factors including reduced production of intracellular reactive oxygen species, increased cellular glutathione, and increased activities of three main endogenous antioxidant enzymes.

## 1. Introduction

Health benefits associated with the consumption of grains are not only linked to the presence of phytochemicals such as polyphenols but also to their content of fibers and micronutrients [1]. The mechanism of protection can be through the reduction of blood cholesterol/glucose, or the prevention of oxidative damage to biomolecules [1,2,3]. Many studies have then focused on the potential of chemicals in grains to prevent or attenuate oxidative stress. It was found, for example, in men with coronary artery disease, that the supplementation of their diets with whole grains (brown rice and barley) resulted in lower oxidation of plasma lipids [1]. Rats fed red and black rice also experienced less renal tubular lipid oxidative damage caused by ferric nitrilotriacetate [2]. Other studies found that oats consumed in the form of oatmeal, oat gum, or oat bran reduced blood low-density lipoproteins cholesterol by 2–23% in both healthy and hypercholesterolemic humans [3,4]. Similarly, high glucan oat brans lowered postprandial plasma glucose and insulin levels in type 2 diabetic and healthy individuals [5]. Benefits of consuming oat products have mainly been attributed to the presence of dietary fibers, phenolic acids and a unique group of amide derivatives known as avenanthramides [6].

Recent studies have, however, shown that, in addition to fibers and polyphenols, hydrolyzed proteins and peptides also contribute to the health promoting effect of cereals [7,8]. Lunasin, a peptide originally discovered in soy and later found in oat, wheat, and barley, demonstrated good radical scavenging and anti-carcinogenic activities in mammalian cells [7]. Protein hydrolysates from oats, wheat, and rice have all shown antioxidant activities against common reactive oxygen species (ROS) in various systems [9,10,11]. The peroxyl radical scavenging activity of oat proteins in the oxygen absorbance capacity assay was found to increase from 53 to 243 µM Trolox equivalents/g after hydrolysis with alcalase [12]. Although major peptides were identified, their activity has not been reported. The aim of the present work was therefore to investigate the radical scavenging activity of those peptides and to determine whether they can regulate oxidative stress in a hepatic cell culture model.

## 2. Materials and Methods

### 2.1. Reagents

Reduced L-glutathione (GSH), fatty acid free bovine serum albumin (BSA), potassium phosphate, 6-hydroxy-2,5,7,8-tetramethylchroman-2-carboxylic acid (Trolox), diethylenetriaminepentaacetic acid (DETAPAC), 3-(4,5-dimethylthiazol-2-yl)-2,5-diphenyltetrazolium bromide (MTT), dimethyl-sulfoxide (DMSO), sodium azide (NaN_3_), glutathione reductase (GR), nicotinamide adenine dinucleotide phosphate (NADPH), cumene hydroperoxide, catalase, nitroblue tetrazolium chloride (NBT), xanthine oxidase, 5,5′-dithiobis-(2-nitrobenzoic acid) (DTNB), 5-sulfosalicylic acid dehydrate, and 96-well and 60-mm tissue culture plates were purchased from Sigma Aldrich (Oakville, ON, Canada). Hydrogen peroxide (H_2_O_2_) and fluorescein were from Fisher Scientific Co., (Nepean, ON, Canada). Bathocuproine disulfonic acid (BCS) and xanthine were purchased from MP Biomedical (Solon, OH, USA) while 2,2′-azobis(2-methylpropionamidine) dihydrochloride (AAPH) was from Wako Chemicals USA Inc., (Richmond, VA, USA). Peptides: FNDRLRQGQLL (P1), GLVYIL (P2), GQTV (P3), GQTVFNDRLRQGQLL (P4), YHNAP (P5), YHNAPGLVYIL (P6), and DVNNNANQLEPR (P7) were synthesized by GenScript (Piscataway, NJ, USA) at a purity of more than 95%.

### 2.2. Oxygen Radical Absorbance Capacity (ORAC)

The ORAC assay was performed as previously described [13,14]. The decay of fluorescein (0.08 µM) by AAPH (150 mM) at 37 °C was recorded at 1 min intervals over 50 min using a BioTek™ FL × 800™ fluorescent microplate reader (Fisher Scientific, Nepean, ON, Canada) with fluorescence filters (excitation 485/20 nm, emission 528/20 nm). Potassium phosphate buffer (pH 7.4, 75 mM) was used to dissolve peptides (100 and 200 µM) and Trolox standards (6.25–100 µM). Data analysis was done with Gen5^TM^ software (Fisher Scientific, Nepean, ON, Canada), and ORAC values were expressed as µM Trolox Equivalent (TE)/µM peptide using the standard curve.

### 2.3. Cell Culture, Cytotoxicity and Cytoprotective Assays

Human hepatocellular carcinoma (HepG2) cells were purchased from the American Type Culture Collection (ATCC; Manassas, VA, USA). They were cultured at 37 °C in a humidified incubator with 5% CO_2_ and 95% air. Dulbecco’s Minimal Essential Media (DMEM) supplemented with 10% (v/v) Fetal Bovine Serum was used for the maintenance of cells. Initial treatment with 20–500 µM of P2 (highest peroxyl radical scavenging activity) showed that 50 and 100 µM were optimum concentrations and both were used for all peptides. The MTT (3-(4,5-dimethylthiazol-2-yl)-2,5-diphenyltetrazolium bromide) assay, as described in an earlier research, was used for cytotoxicity and cytoprotective tests [15]. For both assays, HepG2 cells plated at 2 × 10^4^ cells/well in 96-well tissue culture plates were grown for 24 h, washed with phosphate buffer saline solution (PBS, pH 7.2) followed by 24 h incubation with peptides and another wash, 200 µL of media were then added to cells intended for cytotoxicity and 200 µL of media containing 20 mM of AAPH to those used for cytoprotection evaluation. Following another 24 h incubation, cells were washed twice with 200 µL of PBS. Ten µL of MTT solution (5 mg/mL in PBS) and 100 µL of media were added to each well. After 1 h, MTT solution was removed, and 50 µL DMSO were added. Absorbances were recorded at 570 nm with 630 nm background subtraction using a BioTek Epoch™ microplate reader (Fisher Scientific, Nepean, ON, Canada). Untreated cells were used as negative control (NC) and cells treated with only AAPH were used as positive control (PC). Four peptides (P1, P2, P6, P7) that displayed cytoprotective effect were used for further investigation.

### 2.4. Preparation of Cells Extracts

Cells were seeded at 1 × 10^6^ cells/plate in 60 mm tissue culture plates and allowed to grow for 24 h. In each plate, cells were washed twice with 4 mL of PBS (pH 7.2), and treated with 4 mL peptide samples (P1, P2, P6, P7) dissolved in culture media at 50 or 100 µM for 24 h. After removal of peptide solutions, cells were washed twice with 4 mL of PBS/plate and treated with 4 mL of 20 µM AAPH dissolved in culture media and allowed to incubate for 24 h. Cells in each culture plate were harvested by incubation (5 min) with 0.5 mL of 0.25% trypsin, addition of 1 mL of culture media and centrifugation at 1000× *g* for 5 min. Cell pellets were washed with ice cold PBS (500 µL each) until no pink color was observed, re-suspended in ice cold PBS (300 µL) for the superoxide dismutase (SOD) assay, 500 µL for glutathione peroxidase (GPx) and catalase (CAT) assays. For total glutathione assay, 300 µL of 5% ice cold sulfosalicylic acid bubbled with 100% nitrogen was used for cell lysis. Cells were lysed by sonication on ice for 1 min using a probe-type sonicator (Vibra-Cell, Sonics and Materials Inc., Newtown, CT, USA) pulsing at 15 s on and 10 s off cycles. Following centrifugation of cell lysate at 13,000× *g* (4 °C), pellets containing cell debris were discarded, while supernatants were used for determination of glutathione content. Protein contents were determined using a modified Lowry assay.

### 2.5. Determination of the Activity of Antioxidant Enzymes

The activity of GPx was determined as previously described in a 96-well plate [16]. Briefly, in each well, 187.5 µL of potassium phosphate buffer was mixed with 12.5 µL NADPH (4 mM) solution and 25 µL of cell lysates or buffer (blank), and incubated at 30 °C for 5 min. Then, 100 µL of 0.15% cumene hydroperoxide was added in each well to begin the reaction. The rate of disappearance of NADPH was followed at 340 nm for 5 min. The activity was expressed as Units of GPx activity/mg protein.

The SOD activity was measured based on the rate of reduction of nitroblue tetrazolium (2.24 mM) to formazan by xanthine oxidase (13.2 U/mL) at 560 nm [17]. Eight concentrations of each cell extract (2–500 mg protein/mL) or SOD standard (2–500 ng/mL) were used to obtain inhibition curves. Specifically, 20 µL of cell sample, or buffer (blank) was mixed with 160 µL of assay solution, and 20 µL of xanthine oxidase was added to initiate the reaction. The reduction of nitroblue tetrazolium to blue formazan by O_2_^−•^ generated in situ was followed at 560 nm. SOD activities were calculated and expressed as units per milligram of protein.

To measure the catalase activity, 1790 µL potassium phosphate buffer (50 mM, pH 7.0) was mixed with 200 µL cell lysate in a UV cuvette. Ten µL of H_2_O_2_ (30%) was added, and removal of H_2_O_2_ by catalase was followed at 240 nm [18] using a Cary 50 Bio UV-Vis spectrophotometer with 18-cell changer (Varian Inc., Mississauga, ON, Canada). The activity was expressed as a percentage of control.

### 2.6. Determination of Cellular Glutathione and Reactive Oxygen Species (ROS)

The assay was based on the enzymatic recycling method adjusted for 96-well plates [19]. All solutions were prepared in sodium phosphate buffer (125 mM, pH 7.5) that contained 6.3 mM EDTA (ethylenediaminetetraacetic acid). In summary, 60 µL of 0.35 mM NADPH and 10 µL of 6 mM DTNB were added to wells, this was followed by the addition of 20 µL cell lysate or glutathione (GSH) standard. To initiate the reaction, 10 µL of glutathione reductase (5 IU/mL) was added and the rate of reaction between GSH from cell lysate and DTNB was measured at 412 nm. Total glutathione of cell lysates was calculated based on a standard curve of glutathione.

The measurement of intracellular ROS was determined using a method described by Wolfe and Rui, with modifications [20]. HepG2 cells were seeded at 4 × 10^4^ cells/well in a 96-well tissue culture plate and were treated for 24 h with 100 µM of peptide dissolved in media, with the exception of NC and PC controls. Media was removed, cells washed twice with PBS, incubated (24 h) with 20 mM AAPH and washed again with PBS. Two hundred µL of 40 µM DCFH_2_-DA dissolved in buffer was added, and fluorescent intensity was immediately recorded at 2 min intervals for a total of 60 min. The percentage increase in fluorescent intensity was calculated as shown in Equation (1).

(1) $$Fluorescent\;increase\;\left(\%\right)=\frac{Final\;reading-Initial\;reading}{Initial\;reading}\times 100$$

### 2.7. Statistical Analysis

All data were expressed as means (*n* = 4) ± standard error of mean (S.E.M.). Comparison between groups were carried out by one-way analysis of variance using SPSS 11.0 for Windows (SPSS Inc., Chicago, IL, USA). Post-hoc Tukey’s honest (HSD) test was used to determine significant differences (*p* < 0.05).

## 3. Results

### 3.1. Oxygen Radical Absorbance Capacity (ORAC)

**Figure 1:**
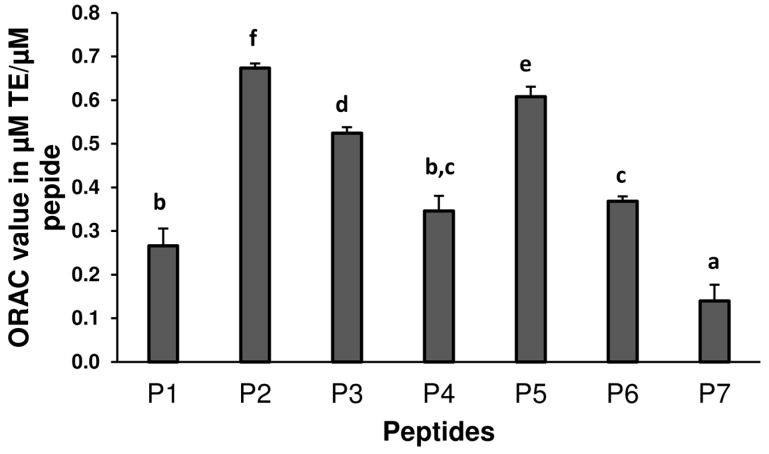
Oxygen radical absorbance capacity (ORAC) values peptides (µmol Trolox equivalent/µmol peptide). FNDRLRQGQLL (P1), GLVYIL (P2), GQTV (P3), GQTVFNDRLRQGQLL (P4), YHNAP (P5), YHNAPGLVYIL (P6), and DVNNNANQLEPR (P7). Each peptide was tested in triplicate. Data are means ± STDEV. Different letters indicate significant differences (*p* < 0.05) in a post-hoc Tukey’s Honest Significant Differences test.

Peroxyl radical (ROO^•^) scavenging activities of P1–P7 were evaluated using the ORAC assay. Data obtained (Figure 1) showed that P2 was the most active peptide, followed by P5 and P3 with ORAC values of 0.67 ± 0.02, 0.61 ± 0.04, and 0.52 ± 0.01 µM Trolox equivalents (TE)/µM of peptide, respectively. P7 was the least active (0.14 ± 0.04 µM TE/µM). ROO^•^ scavenging activities can be affected by sequences, molecular weights and the presence of amino acids that can form stable radical intermediates after donation of electrons or protons [14]. The highest activities of P2 and P5 can be explained by the presence in their sequences of both tyrosine (Y), an aromatic amino acid that can easily donate a proton and leucine (L) that can enhance hydrophobic interactions as suggested in a previous study [21]. P3 had no aromatic ring moiety, but contained threonine whose hydroxyl group on the side chain might have enhanced its activity. Although P5 contained an additional radical stabilizing amino acid, histidine (H), its activity was lower than that of P2, emphasizing the importance of an optimum sequence for antioxidant protection. On a mass basis, ORAC values of P1–P7 ranged from 190.7–603.8 µM TE/g peptide compared to 242.5 µM TE/g reported in the literature for the hydrolysate from which they were identified [12]. Other studies have reported greater antioxidant activity for low molecular weight peptides (2–10 amino acids) and it has been speculated that it was due to their ease of access to peroxyl radicals [22]. The ORAC values of P1, P4, and P6 were similar to the activity MHIRL from beta-lactoglobulin (0.306 µM TE/µM) but lower than WYSLAMAASDI (2.6 µM/µM) from the same protein [23] or YAEERYPIL from egg white (3.5 µM TE/µM) [24].

### 3.2. Cytotoxicity and Cytoprotective Effects of Peptides

**Figure 2:**
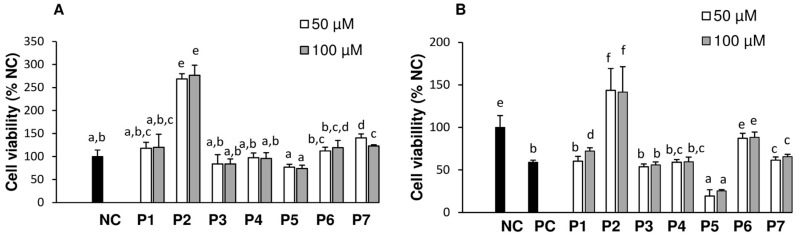
Cytotoxicity and cytoprotective effects of the tested peptides on HepG2 cells: NC (negative control), PC (positive control), FNDRLRQGQLL (P1), GLVYIL (P2), GQTV (P3), GQTVFNDRLRQGQLL (P4), YHNAP (P5), YHNAPGLVYIL (P6), and DVNNNANQLEPR (P7). For cytotoxicity (**A**) Cells were treated with peptides for 24 h and for cytoprotection (**B**) cells were pretreated with peptides for 24 h followed by 24 h incubation with 20 mM 2,2′-azobis(2-methylpropionamidine) dihydrochloride (AAPH). Negative control (NC) cells were not treated with peptide or APPH while positive control (PC) cells were treated with AAPH only. Data are means ± SEM. Different letters indicate significant differences (*p* < 0.05) in a post-hoc Tukey’s Honest Significant Differences test.

The cytotoxicity of each peptide was determined by measuring the viability of HepG2 cells. The assay was initially optimized using P2 (20–500 µM) because of its highest activity in the ORAC assay. It was only cytotoxic above 450 µM. Subsequent tests were then performed using 50 and 100 µM only. As shown in Figure 2A, treatment with P1, P3, P4, P5, P6, and P7 did not affect HepG2 cells viability (*p* > 0.05) compared to that of NC. It was found that P2 greatly promoted the growth of HepG2 cells as evidenced by the nearly 2.7-fold increase in cell viability. The mechanism is unclear but similar findings were reported in hamster ovary cells treated with yeast, soy, and broadbean hydrolysates [8]. The growth enhancement of P2 might be due to its greater uptake as suggested for endothelial cells treated with alpha-tocopherol [25].

Cytoprotective effects of peptides against oxidant-induced damages (Figure 2B) showed that HepG2 cells treated with APPH only (PC) experienced a 40% decrease in viability compared to negative control (NC, no APPH). Peptides P3 and P4 had no effect on oxidative damages while P1 and P7 at 100 µM were partly protective (*p* < 0.05) as they increased the viability of cells by 15.5% and 8.3%, respectively, compared to PC. Pretreatment of cells with P6 increased the viability of cells from 58.9% ± 2.7% for PC to 88.2% ± 6.4%. The most cytoprotective peptide was P2, which not only completely eliminated AAPH damage, but also increased the viability of HepG2 cells, compared to NC. The protective effect of P2 is partly due to the fact that in the absence of AAPH, it did increased cell viability (Figure 2A). The cytoprotection P2 > P6 > P1 ≈ P7 was found to correlate with their degree of hydrophobicity, but not with ORAC values. In addition to hydrophobicity, the positive charge of P1 and P6 may have enhanced their interaction with membrane phosphorus groups. P6 also contains histidine that can protect cells through its metal binding capability, as suggested for bean protein hydrolysates in Caco-2 cells [26]. It is possible that the low hydrophobicity of P3 and P4 limited their interaction with membrane and subsequently their cellular uptake. Although P2 was the most active peptide in both assays, P5 which was the second most active in the ORAC test, enhanced cytotoxicity in cells. In fact, some molecules with antioxidant capacity in vitro have been shown to act as pro-oxidants in vivo, causing apoptosis as a result of mitochondrial dysfunction or DNA damage [27,28].

### 3.3. Activity of Antioxidant Enzymes

Four of the peptides P1, P2, P6 and P7 showed cytoprotective properties at either 50 or 100 µM. In order to elucidate the possible mechanism, their ability to regulate the activity of three main anti-oxidative enzymes, decrease intracellular production of ROS or regulate glutathione were determined. AAPH is an oxidation inducer that generates peroxyl radicals by thermal decomposition at a constant rate and is believed to cause damage primarily to membrane lipids and proteins [29]. The oxidized lipids or proteins in the form of hydroperoxides can be reduced to alcohol derivatives by GPx. Oxidants can also transfer electrons to molecular oxygen generating superoxide anion radicals which are also responsible for oxidative damage to biomolecules. The oxidized oxygen can be converted by SOD to less reactive hydrogen peroxide which can be reduced to water mainly by catalase. Regulating the activity of these enzymes will then have an influence on oxidative stress.

**Figure 3:**
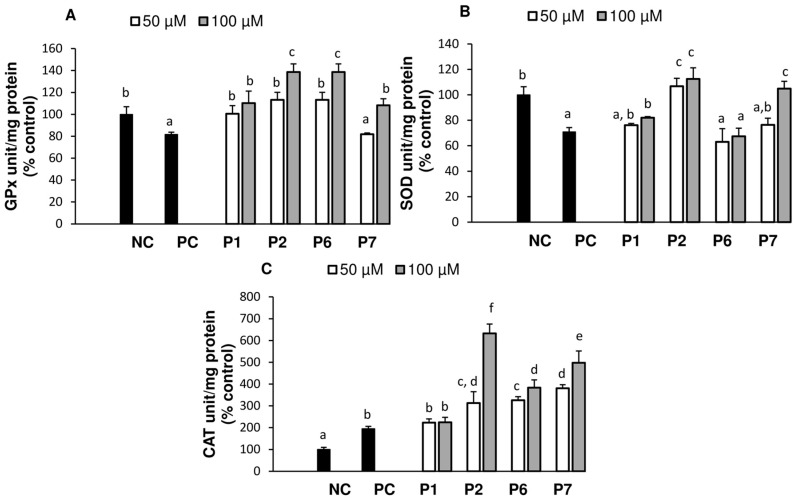
Effect of pre-treatment of AAPH-stressed HepG2 cells with peptides P2 and P4–P7 (0, 50 or 100 µM) on the activity of glutathione peroxidase (GPx, **A**), superoxide dismutase (SOD, **B**), and catalase (CAT, **C**). NC (negative control), PC (positive control). Data are expressed as means (*n* = 3) ± SEM and significant differences (*p* < 0.05, One-way ANOVA) in post-hoc Tukey’s Honest Significant Differences test are indicated by different letters).

As shown in Figure 3A, incubation of HepG2 cells with AAPH decreased GPx activity by 18.2%, relative to NC. Pre-treatment with P1, P2, and P6 at 50 µM completely restored the activity while pre-treatment with P7 (50 µM) had no effect. At 100 µM, P2 and P6 further increased GPX activity (*p* < 0.05) above that of normal cells by 30.2% and 38.6%, respectively, while P7 only suppressed the damage caused by AAPH. These data demonstrated that higher cytoprotective effects for P2 and P6 in HepG2 cells might be due to their ability to detoxify hydroperoxides, as they are the main oxidative products of AAPH [29]. In the SOD assay, (Figure 3B) P1 and P7 at 50 µM, P6 (50 and 100 µM) did not affect the activity compared to AAPH treated cells only (PC). P2 (50 and 100 µM) was able to increase SOD to a level equivalent to that of normal cells. P6, which had higher cytoprotection and elevation of GPx level, did not affect SOD activity. It appeared that the tested peptides only slightly affected SOD in comparison to a much larger effect on the activity of GPx. Since SOD acts on the superoxide anion radical and GPx on hydroperoxides, it is logical to conclude that the damage to HepG2 is mainly attributed to lipid or protein hydroperoxides generated in the presence of AAPH. Data from the catalase (CAT) assay (Figure 3C) showed that treatment with AAPH alone increased the CAT activity by almost 2-fold. Pre-treatment with each peptide further upregulated CAT activity level compared to PC cells. Specifically, with 50 µM, the activity was further increased by 60% for P2 and P6; and 90% for P7. Meanwhile, no change was observed for P1. At 100 µM, the CAT activity of P2 almost doubled while P1 was not affected by the peptide concentration.

Compared to SOD and GPx, CAT is resistant to inactivation by peroxyl radicals, due to the narrowness of its active site which prevents access of large molecules [18]. The increase of CAT activity in this work, for APPH treated cells, might be due to an induction by lipid peroxides as reported for smooth muscle, macrophage and umbilical vein endothelial cells [30]. Oxidants such as H_2_O_2_ were also reported to double CAT activity of rat hepatoma cells while pretreatment with epicatechin tripled the value [31]. Peptides from fish skin gelatin hydrolysate increased SOD, GPx, and CAT activities in hepatic Hep3B cells by up to 92.8% [16]. In porcine kidney epithelial cells [32], the decrease in activities of GPx and SOD in the presence of AAPH was attributed to the oxidation of tryptophan and histidine at their respective active site [33,34]. Hence, the tested peptides may have protected those sites.

### 3.4. Intracellular Reactive Oxygen Species and Glutathione

**Figure 4:**
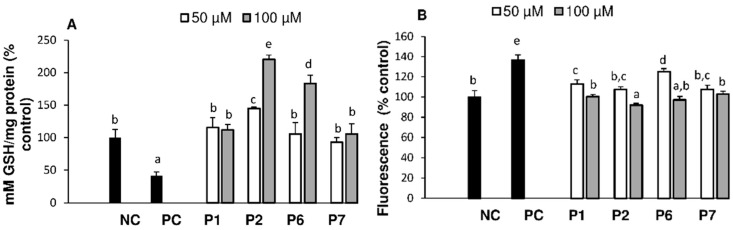
Glutathione (GSH, **A**) and reactive oxygen species (ROS, **B**) levels of HepG2 cells pre-treated with peptides P2, P4–P7 (0, 50, 100 µM) followed by AAPH-induced stress. Data are means ± SEM. Significant differences in post-hoc Tukey’s Honest Significant Differences (HSD) test are represented by different letters (*p* < 0.05, One-Way ANOVA).

The physiological role of GSH has long been of interest, particularly its antioxidant function due to the unique redox chemistry of its cysteinyl thiol residue [35]. The oxidation of sulfhydryl groups on amino acids by ROS can lead to the formation of protein disulfide bonds, which can be reduced by GSH into individual sulfhydryl groups. GSH is also a substrate used by GPx in the reduction of peroxides and its concentration often reflects oxidative status of cells. Treatment with AAPH decreased GSH by 58.1% (Figure 4A) which was eliminated by pre-treatment of cells with all four peptides at 50 µM, while at 100 µM, P2 and P6 further increased (*p* < 0.05) GSH concentrations above that of normal cells. It has been found that in HepG2 cells, reduced glutathione (GSH) is approximately 30-fold higher than its oxidized form GSSG [36]. In the present study, the concentration of GSSG was below the detection limit. The decrease of GSH observed in the present study after AAPH treatment might be due to its direct radical scavenging properties because of a concomitant decrease in intracellular ROS, as measured by the Dichlorofluorescin (DCF) assay (Figure 4B). In the presence of excess oxidant, GSH can form mixed disulfide bonds and precipitate with the protein fraction prior to the assay, resulting in lower concentrations as well. The tested peptides, by acting as scavengers, maintained higher cellular GSH concentration. Other studies also found that the increase of ROS due to AAPH was attenuated after treatment with peptides VCSV and CAAP from fish, and tilapia hydrolysate in rat macrophage RAW 264.7 cells [37] or HepG2 cells [38].

## 4. Conclusions

Results from this study showed that peptides had various scavenging activities in the ORAC assay with P2, P5 and P3 being the most active. Individual peptides alone did not show cytotoxicity in HepG2 cells. However, when followed by AAPH induced oxidative stress, P5 enhanced cells death while P1, P2, P6, and P7 were cytoprotective. The mechanism of protection, particularly for P2 and P6, was related to the increased activities of antioxidant enzymes GPx, SOD and CAT; increase in glutathione synthesis, and reduced production of intracellular reactive oxygen species.

## References

[1] Jang Y., Lee J.H., Kim O.Y., Park H.Y., Lee S.Y. (2001). Consumption of Whole Grain and Legume Powder Reduces Insulin Demand, Lipid Peroxidation, and Plasma Homocysteine Concentrations in Patients With Coronary Artery Disease: Randomized Controlled Clinical Trial. Arterioscler. Thromb. Vasc. Biol..

[2] Toyokuni S., Itani T., Morimitsu Y., Okada K., Ozeki M., Kondo S., Uchida K., Osawa T., Hiai H., Tashiro T. (2002). Protective Effect of Colored Rice over White Rice on Fenton Reaction-based Renal Lipid Peroxidation in Rats. Free Radic. Res..

[3] Wu Y., Qian Y., Pan Y., Li P., Yang J., Ye X., Xu G. (2015). Association between dietary fiber intake and risk of coronary heart disease: A meta-analysis. Clin. Nutr..

[4] Van Horn L., Emidy L.A., Liu K.A., Liao Y.L., Ballew C., King J., Stamler J. (1988). Serum lipid response to a fat-modified, oatmeal-enhanced diet. Prev. Med..

[5] Braaten J.T., Scott F.W., Wood P.J., Riedel K.D., Wolynetz M.S., Brulé D., Collins M.W. (1994). High beta-glucan oat bran and oat gum reduce postprandial blood glucose and insulin in subjects with and without type 2 diabetes. Diabet. Med..

[6] Liu S., Yang N., Hou Z.H., Yao Y., Lü L., Zhou X.R., Ren G.X., Lu L. (2011). Antioxidant Effects of Oats Avenanthramides on Human Serum. Agric. Sci. China.

[7] Nakurte I., Kirhnere I., Namniece J., Saleniece K., Krigere L., Mekss P., Vicupe Z., Bleidere M., Legzdina L., Muceniece R. (2013). Detection of the lunasin peptide in oats (*Avena sativa* L). J. Cereal Sci..

[8] Lee J.Y., Chun B.H., Lee J.H., Ahn J., Chung N. (2009). Influence of Mixed Protein Hydrolysates on the Growth and Viability of Chinese Hamster Ovary Cells. J. Korean Soc. Appl. Biol. Chem..

[9] Handelman G.J., Cao G., Walter M.F., Nightingale Z.D., Paul G.L., Prior R.L., Blumberg J.B., Blumberg J.B. (1999). Antioxidant capacity of oat (*Avena sativa* L.) extracts. 1. Inhibition of low-density lipoprotein oxidation and oxygen radical absorbance capacity. J. Agric. Food Chem..

[10] Zhu K., Zhou H., Qian H. (2006). Antioxidant and free radical-scavenging activities of wheat germ protein hydrolysates (WGPH) prepared with alcalase. Process. Biochem..

[11] Nam S.H., Choi S.P., Kang M.Y., KozUKue N., Friedman M. (2005). Antioxidative, antimutagenic, and anticarcinogenic activities of rice bran extracts in chemical and cell assays. J. Agric. Food Chem..

[12] Jodayree S., Smith J.C., Tsopmo A. (2012). Use of carbohydrase to enhance protein extraction efficiency and antioxidative properties of oat bran protein hydrolysates. Food Res. Int..

[13] Huang D., Ou B., Hampsch-Woodill M., Flanagan J.A., Prior R.L. (2002). High-throughput assay of oxygen radical absorbance capacity (ORAC) using a multichannel liquid handling system coupled with a microplate fluorescence reader in 96-well format. J. Agric. Food Chem..

[14] Tsopmo A., Diehl-Jones B.W., Aluko R.E., Kitts D.D., Elisia I., Friel J.K. (2009). Tryptophan released from mother’s milk has antioxidant properties. Pediatr. Res..

[15] Nguyen K.C., Rippstein P., Tayabali A.F., Willmore W.G. (2015). Mitochondrial Toxicity of Cadmium Telluride Quantum Dot Nanoparticles in MamMalian Hepatocytes. Toxicol. Sci..

[16] Mendis E., Rajapakse N., Kim S.K. (2005). Antioxidant properties of a radical-scavenging peptide purified from enzymatically prepared fish skin gelatin hydrolysate. J. Agric. Food Chem..

[17] Spitz D.R., Oberley L.W. (1989). An assay for superoxide dismutase activity in mammalian tissue homogenates. Anal. Biochem..

[18] Pigeolet E., Corbisier P., Houbion A., Lambert D., Michiels C., Raes M., Zachary M.D., Remacle J. (1990). Glutathione peroxidase, superoxide dismutase, and catalase inactivation by peroxides and oxygen derived free radicals. Mech. Ageing Dev..

[19] Tietze F. (1969). Enzymic method for quantitative determination of nanogram amounts of total and oxidized glutathione: Applications to mamMalian blood and other tissues. Anal. Biochem..

[20] Wolfe K.L., Rui H.L. (2007). Cellular antioxidant activity (CAA) assay for assessing antioxidants, foods, and dietary supplements. J. Agric. Food Chem..

[21] Byun H.G., Lee J.K., Park H.G., Jeon J.K., Kim S.K. (2009). Antioxidant peptides isolated from the marine rotifer, Brachionus rotundiformis. Process. Biochem..

[22] Ranathunga S., Rajapakse N., Kim S.K. (2006). Purification and characterization of antioxidative peptide derived from muscle of conger eel (*Conger myriaster*). Eur. Food Res. Technol..

[23] Hernández-Ledesma B., Dávalos A., Bartolomé B., Amigo L. (2005). Preparation of antioxidant enzymatic hydrolysates from α-lactalbumin and β-lactoglobulln. Identification of active peptides by HPLC-MS/MS. J. Agric. Food Chem..

[24] Davalos A., Miguel M., BartoLome B., Lopez-Fandino R. (2004). Antioxidant activity of peptides derived from egg white proteins by enzymatic hydrolysis. J. Food Prot..

[25] Kuzuya M., Naito M., Funaki C., Hayashi T., Yamada K., Asai K., Kuzuya F. (1991). Antioxidants stimulate endothelial cell proliferation in culture. Artery.

[26] Carrasco-Castilla J., Hernández-Álvarez A.J., Jiménez-Martínez C., Jacinto-Hernández C., Alaiz M., Girón-Calle J., Vioque J., DáVila-Ortiz G. (2012). Antioxidant and metal chelating activities of peptide fractions from phaseolin and bean protein hydrolysates. Food Chem..

[27] Azmi A.S., Bhat S.H., Hadi S.M. (2005). Resveratrol-Cu(II) induced DNA breakage in human peripheral lymphocytes: Implications for anticancer properties. FEBS Lett..

[28] Galati G., O’Brien P.J. (2004). Potential toxicity of flavonoids and other dietary phenolics: Significance for their chemopreventive and anticancer properties. Free Radic. Biol. Med..

[29] He R.R., Li Y., Li X.D., Yi R.N., Wang X.Y., Tsoi B., Lee K.K.H., Abe K., Yang X., Kurihara H. (2013). A New Oxidative Stress Model, 2,2-Azobis(2-Amidinopropane) Dihydrochloride Induces Cardiovascular Damages in Chicken Embryo. PLoS ONE.

[30] Meilhac O., Zhou M., Santanam N., Parthasarathy S. (2000). Lipid peroxides induce expression of catalase in cultured vascular cells. J. Lipid Res..

[31] Roig R., Cascón E., Arola L., Bladé C., Salvadó M.J. (2002). Procyanidins protect Fao cells against hydrogen peroxide-induced oxidative stress. Biochim. Biophys. Acta..

[32] Park M.-J., Han J.-S. (2008). Fucoidan protects LLC-PK1 cells against AAPH-induced damage. J. Food Sci. Nutr..

[33] Epp O., Ladenstein R., Wendel A. (1983). The refined structure of the selenoenzyme glutathione peroxidase at 0.2-nm resolution. Eur. J. Biochem..

[34] Johnson M.A., Macdonald T.L. (2004). Accelerated CuZn-SOD-mediated oxidation and reduction in the presence of hydrogen peroxide. Biochem. Biophys. Res. Commun..

[35] Cotgreave I.A., Gerdes R.G. (1998). Recent trends in glutathione biochemistry—glutathione-protein interactions: A molecular link between oxidative stress and cell proliferation?. Biochem. Biophys. Res. Commun..

[36] Vidyashankar S., K Mitra S., Nandakumar K.S. (2010). Liv.52 protects HepG2 cells from oxidative damage induced by tert-butyl hydroperoxide. Mol. Cell. Biochem..

[37] Ko J.Y., Lee J.H., Samarakoon K., Kim J.S., Jeon Y.J. (2013). Purification and determination of two novel antioxidant peptides from flounder fish (*Paralichthys olivaceus*) using digestive proteases. Food Chem. Toxicol..

[38] Yarnpakdee S., Benjakul S., Kristinsson H.G., Bakken H.E. (2015). Preventive effect of Nile tilapia hydrolysate against oxidative damage of HepG2 cells and DNA mediated by H_2_O_2_ and AAPH. J. Food Sci. Technol..

